# Palinopsia as an initial symptom of cerebral amyloid angiopathy-related inflammation

**DOI:** 10.1016/j.ensci.2021.100375

**Published:** 2021-10-28

**Authors:** Hitomi Onomura, Takahiro Shimizu, Rei Kobayashi, Junichiro Suzuki, Noriyoshi Nakai, Satoshi Okuda, Yasuhiro Ito

**Affiliations:** a Department of Neurology, Toyota Memorial Hospital, 1-1 Heiwa-cho, Toyota, Aichi 471-8513, Japan; b Department of Neurology, National Hospital Organization Nagoya Medical Center, Nagoya, 4-1-1Sannomau, Naka-ku, 460-0001, Nagoya, Japan; c Department of Neurology, National Hospital Organization Higashinagoya National Hospital, Nagoya, 5-101 Umamorizaka, Meito-Ku, 465-8550, Nagoya, Japan

**Keywords:** Palinopsia, Amyloid beta related inflammation, Microbleeds, Suspected weighted image, Central nervous system vasculitis, Visual preservation, Cerebral amyloid angiopathy

## Abstract

• We report the first case of cerebral amyloid angiopathy-related inflammation (CAA-RI) presenting palinopsia initially.
• Palinopsia is generally caused by intracranial diseases involving the parietal and occipital areas.
• CAA dominantly affects parietal and occipital lobes, therefore palinopsia could be an important phenomenon of the disease.

Dear Editor,

Palinopsia refers to the persistence or recurrence of a visual image after the removal of visual stimuli. Most cases presenting palinopsia are reported to be caused by seizures, tumors, ischemic or hemorrhagic cerebrovascular disease involving the parietal area [1]. Here we report a case of cerebral amyloid angiopathy-related inflammation (CAA-RI) presenting palinopsia as an initial symptom. Furthermore, we present a brief review of the literature in relation to this clinical case. The patient is a 60-year-old right-handed man with a history of hypertension. While watching a man in a video, he moved his viewpoint on a wall nearby and noticed that the afterimage of the man's face remained visible and moving. Symptoms also occurred with smartphone screens, he struggled to operate his phone after staring at the screen, because the image duplicated next to the original. Images were either all or part of an object that he had seen just before and repeated the preceding motion. For example, he saw a speaking mouth on a wall right after talking to anyone and staring at their mouths. Images mostly appeared at the right half side of his visual field and lasted several tens of seconds to a few minutes. It recurred for several days from onset, whenever he stared at a new object. He refused to look at anything around him and was prone to close his eyes. On presentation, his blood pressure was high, 175/121 mmHg. Physical and neurological examination revealed right unilateral spatial neglect on the line bisection task, but other findings were within normal limits. Mini-mental scale examination was full (30/30). Screening bloodwork and extensive hematologic autoimmune disease work-up were negative. The genotype of apolipoprotein E was ε3/ε3. Ophthalmological evaluation including fundoscopy, tonometry and anterior chamber examination was normal. FLAIR sequence on brain magnetic resonance imaging (MRI) showed white matter hyperintensity in the occipital and parietal lobes predominantly affecting the left side, suggesting cerebral edema. The superficial vessels in the corresponding areas were enhanced by gadolinium-contrasted media, susceptive of vascular inflammation. T2* image and susceptibility-weighted imaging (SWI) revealed multiple cortical microbleeds (MBs) in the same area, but neither cortical superficial siderosis (cSS) nor convexity subarachnoid hemorrhage (cSAH) were recognized (Fig. 1). Technetium-99m ethyl cysteinate dimer (99mTc ECD) single photon emission computed tomography (SPECT) revealed reduced blood flow in the left occipital lobe (Fig. 1B). Cerebrospinal fluid examination presented both cell and protein count within normal range, neither of IgG, IgA, IgM was elevated. Electroencephalography presented normal findings, with a 12 Hz alpha wave. These results met the diagnostic criteria for probable CAA-RI [2]. Treatment for high blood pressure was initiated with amlodipine besylate and olmesartan medoxomil on day 3. Blood pressure rapidly improved around to 120/80 mmHg average; however, brain edema and inflammation persisted, and he still complained of palinopsia. Oral prednisolone at 1 mg/kg was started on day 10, tapered off thereafter by decrements of 10 mg every 2 weeks, until a maintenance dose of 5 mg daily. Palinopsia resolved promptly, and recurrence of any other symptoms was not observed. Abnormal MRI findings including cerebral edema and gadolinium-enhanced vessels gradually improved, except for the MBs in SWI (Fig. 1C). SPECT findings also gradually recovered. Recurrence has not been observed after 9 months of follow up.

**Fig. 1:**
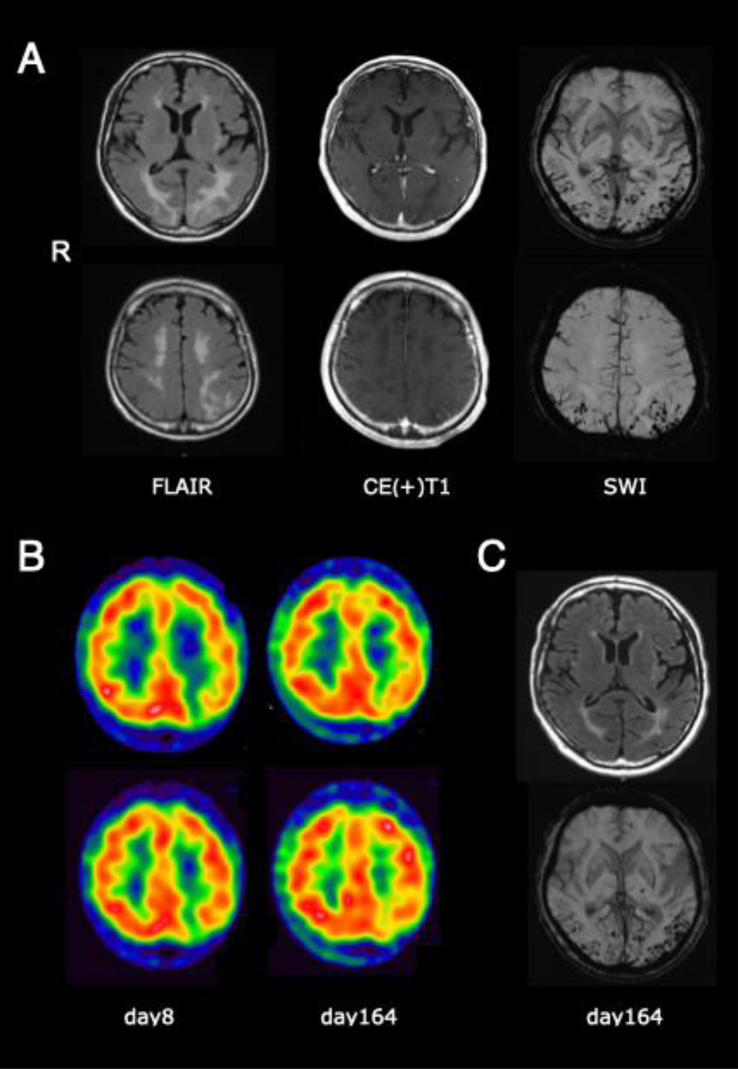
A. Head MRI fluid attenuated inversion recovery sequence (FLAIR) presents cerebral edema from the left occipital to parietal lobe. Gadolinium-enhanced contrasted effects of the superficial vessels in the same region. SWI shows multiple cortical MBs in the left occipital and parietal lobes. (B) 99mTc ECD SPECT. Reduced blood flow in the left occipital lobe at day 8, improvement seen at day 164. (C) MRI on day 164. Although FLAIR present increased signals improving under treatment progress, the MBs of SWI remain unchanged.

Our CAA-RI case presented palinopsia as an initial symptom. Palinopsia is classified into two subgroups: hallucinatory and illusionary. The former refers to the afterimage, formed and preserved, isochromatic and in high resolution. The latter has the afterimages unformed, indistinct, with low resolution, affected by ambient light and motion [1]. Our case presents hallucinatory palinopsia. Pathophysiological causes include a fault in the visual stimuli inhibitory system, impairment of the neural function that regulates the inhibitory system, or loss of visual information to elicit inhibitory signals. Furthermore, reports reveal disorders of the parietal and occipital lobes of the hemisphere as causes, more commonly sided in the right than left. An association with the interparietal sulcus between the superior and inferior parietal lobules has also been considered, as it is significant in the retention of visual information for short periods of time [3,4].

CAA is a cerebral small vessel disease, characterized by the progressive accumulation of amyloid beta protein. Amyloid accumulation induces the degeneration, fragility and destruction of the cerebral vessels, resulting in MBs, cSS, cSAH and cerebral lobar hemorrhage in the worst cases [5,6]. These angiopathic changes generally occur posterior dominant. CAA-RI is a specific and subacute form of CAA [7,8]. The precise mechanism remains unknown; however, the immunogenicity of amyloid beta protein deposited in blood vessels is thought to induce inflammatory reactions including immune cell infiltration, brain edema and vasculitis localized to the central nervous system. Immunosuppressive therapy is an effective treatment as in our case. Common clinical symptoms of CAA-RI are subacutely progressive cognitive or functional decline and seizure. Visual disturbance including hemianopsia and hallucinations are also reported [7]; however, we found no previous detailed report of a case presenting palinopsia. Underlying causes of palinopsia in past reports are mainly seizures, space-occupying lesions, and cerebrovascular events involving parietal and occipital area [9]. Considering that parietal and occipital lobes are dominantly affected in CAA, it is reasonably plausible that palinopsia may occur in this disease entity. Moreover, palinopsia is considered as a kind of positive symptom. Transient focal neurological episodes (TFNE) are also well-known neurological symptoms in CAA, including not only negative symptoms such as focal weakness or hypesthesia, but also positive symptoms, such as aura-like or visual phenomena [10]. Symptoms of TFNE are mostly ‘transitory and last only for a few minutes, however in our case, palinopsia recurred for several days. Additionally, although TFNE in CAA tend to accompany cSS or cSAH in neuroimaging our case did not present findings consistent with these specific features. This suggests that the mechanism of palinopsia may somewhat differ from that of TFNE in CAA. Further research is required to determine its detailed mechanism.

## Ethical considerations

Written informed consent was granted by the patient/family/next of kin to publish case details. No ethics committee approval was required.

## Funding sources

This research did not receive any specific grant from funding agencies in the public, commercial, or not-for-profit sectors.

## Data availability statement

The data that support the findings of this study are openly available.

## Declaration of Competing Interest

None.

## References

[1] Gersztenkorn D., Lee A.G. (2015). Palinopsia revamped: a systematic review of the literature. Surv Ophthalmol.

[2] Auriel E., Charidimou A., Gurol M.E., Ni J., Van Etten E.S., Martinez-Ramirez S., Boulouis G., Piazza F., DiFrancesco J.C., Frosch M.P., Pontes-Neto O.V., Shoamanesh A., Reijmer Y., Vashkevich A., Ayres A.M., Schwab K.M., Viswanathan A., Greenberg S.M. (2016). Validation of Clinicoradiological criteria for the diagnosis of cerebral amyloid angiopathy-related inflammation. JAMA Neurol.

[3] Van der Stigchel S., Nijboer T.C., Bergsma D.P., Barton J.J., Paffen C.L. (2012). Measuring palinopsia: characteristics of a persevering visual sensation from cerebral pathology. J Neurol Sci.

[4] Todd J.J., Marois R. (2004). Capacity limit of visual short-term memory in human posterior parietal cortex. Nature.

[5] Charidimou A., Boulouis G., Gurol M.E., Ayata C., Bacskai B.J., Frosch M.P., Viswanathan A., Greenberg S.M. (2017). Emerging concepts in sporadic cerebral amyloid angiopathy. Brain.

[6] Greenberg S.M., Al-Shahi Salman R., Biessels G.J., van Buchem M., Cordonnier C., Lee J.M., Montaner J., Schneider J.A., Smith E.E., Vernooij M., Werring D.J. (2014). Outcome markers for clinical trials in cerebral amyloid angiopathy. Lancet Neurol.

[7] Scolding N.J., Joseph F., Kirby P.A., Mazanti I., Gray F., Mikol J., Ellison D., Hilton D.A., Williams T.L., MacKenzie J.M., Xuereb J.H., Love S. (2005). Abeta-related angiitis: primary angiitis of the central nervous system associated with cerebral amyloid angiopathy. Brain.

[8] Danve A., Grafe M., Deodhar A. (2014). Amyloid beta-related angiitis--a case report and comprehensive review of literature of 94 cases. Semin Arthritis Rheum.

[9] Chung K.K., Anderson N.E., Hutchinson D., Synek B., Barber P.A. (2011). Cerebral amyloid angiopathy related inflammation: three case reports and a review. J Neurol Neurosurg Psychiatry.

[10] Charidimou A., Peeters A., Fox Z., Gregoire S.M., Vandermeeren Y., Laloux P., Jager H.R., Baron J.C., Werring D.J. (2012). Spectrum of transient focal neurological episodes in cerebral amyloid angiopathy: multicentre magnetic resonance imaging cohort study and meta-analysis. Stroke.

